# Association between Prenatal Dietary Toxicants and Infant Neurodevelopment: The Role of Fish

**DOI:** 10.3390/toxics12050338

**Published:** 2024-05-06

**Authors:** Xiruo Kou, Nerea Becerra-Tomás, Josefa Canals, Monica Bulló, Victoria Arija

**Affiliations:** 1 Nutrition and Mental Health (NUTRISAM) Research Group, Universitat Rovira i Virgili, 43204 Reus, Spain; kouxiruo@gmail.com (X.K.); nerea.becerra@urv.cat (N.B.-T.); josefa.canals@urv.cat (J.C.); 2 Institut d’Investigació Sanitaria Pere Virgili (IISPV), 43204 Reus, Spain; monica.bullo@urv.cat; 3 Centre de Recerca en Avaluació i Mesura de la Conducta (CRAMC), Department of Psychology, Universitat Rovira i Virgili, 43007 Tarragona, Spain; 4 University Research Institute on Sustainablility, Climate Change and Energy Transition (IU-RESCAT), Universitat Rovira i Virgili, 43003 Tarragona, Spain; 5 CIBER Physiology of Obesity and Nutrition (CIBEROBN), Carlos III Health Institute, 28029 Madrid, Spain; 6 Center of Environmental, Food and Toxicological Technology—TecnATox, Rovira i Virgili University, 43201 Reus, Spain; 7 Collaborative Research Group on Lifestyles, Nutrition and Smoking (CENIT), Tarragona-Reus Research Support Unit, Jordi Gol Primary Care Research Institute, 43003 Tarragona, Spain

**Keywords:** dietary exposure, food toxicants, pregnant women, infant neurodevelopment, fish

## Abstract

More research is needed to understand how the maternal consumption of fish and fish-borne toxicants impacts infant neurodevelopment. The present analysis was conducted over 460 mother–infant pairs within the ECLIPSES study. Dietary intake of metals and persistent organic pollutants from fish (including white fish, blue fish, and seafood) was estimated in pregnant women. The infants underwent cognitive, language, and motor function assessments using the Bayley Scales of Infant Development-III at the 40-day postpartum. Associations between dietary toxicants and outcomes were assessed using multivariable linear regression models. Estimated prenatal exposure to fish-borne toxicants, such as arsenic, inorganic arsenic, methylmercury, dioxin-like polychlorinated biphenyls (DL-PCBs), and non-DL-PCBs, was associated with poorer language functions in infants, whereas no significant associations were found with motor or cognitive functions. Maternal fish consumption exceeding the Spanish recommendation of no more than 71 g per day was linked to these adverse effects on language abilities without affecting motor or cognitive development. This highlights the importance of vigilant monitoring of environmental toxicants and the provision of dietary guidance for pregnant women, with potential implications for public health and child development.

## 1. Introduction

The fetal stage of neurodevelopment is a critical period that lays the foundation for lifelong neurological function [1,2]. During this sensitive phase, the developing fetus is particularly vulnerable to the adverse effects of environmental toxicants, owing to its ongoing physiological maturation and immature detoxification mechanisms [3,4,5]. As a result, exposure to toxicants during pregnancy is highly related to the neurodevelopment of offspring, with potential implications for their overall health and well-being [6,7].

The exposure of the fetus to toxicants can be influenced by the mother’s dietary intake, both through the accumulation of compounds with long half-lives and through recent consumption. This highlights the significance of maternal diet in determining fetal toxicant levels [8]. Dietary toxicants are now a major concern in the field of environmental toxicants, especially in industrial areas where they have attracted substantial attention [9,10]. In a previous study, we found that daily intake ofarsenic (As) and dioxin-like polychlorinated biphenyls (DL-PCBs) in pregnant women exceeded or came close to European Food Safety Authority (EFSA) thresholds [11]. Notably, a considerable proportion of dietary toxicants was attributed to fish consumption [11]. Maternal fish consumption during pregnancy provides beneficial nutrients like n-3 polyunsaturated fatty acids (n-3 PUFA), proteins, and vitamins for fetuses [12]. However, the substantially high amounts of toxicants could adversely affect fetal growth and neurodevelopment. Of note, the toxicants’ variability within fish species and regions resulted in varying regional dietary guidelines regarding the recommended quantity ranges for fish consumption [13,14,15].

To date, the association between prenatal dietary exposure to toxicants and the neurodevelopment of offspring has been investigated. Prenatal dietary exposure to high methylmercury (MeHg) or dioxins and PCBs has been related to poor language functions among 3-year-old children [16,17]. Similarly, lower levels of prenatal dietary exposure to MeHg (fish intake ≤ 400 g/week) were associated with elevated language and communication functions in 5-year-old children, whereas high exposure (fish intake > 400 g/week) demonstrated the opposite trend [18]. Most studies published currently focus on children rather than infants (from birth to 1 year old) [19], despite the importance of the infant stage, which holds great potential for early intervention. Moreover, estimating dietary intake at only a single time-point during pregnancy rather than across the entire gestational period may limit the ability to capture a holistic representation of the maternal diet [20]. Considering the diverse toxicants that fish contain and the role of early infancy neurodevelopment in shaping a healthy child’s life, there is a crucial need to comprehensively evaluate the association between the maternal dietary intake of toxicants from fish and infant neurodevelopment [11,21].

Thus, this study aims to assess associations between estimated prenatal dietary fish consumption and derived toxicants with the language, motor, and cognitive development of infants at 40 days of age, aiming to provide more insights for potential guidance in public health strategies.

## 2. Materials and Methods

### 2.1. Study Design and Participants

The present analysis was conducted with a subset of subjects within the ECLIPSES study, a community-based research project conducted among pregnant women in the province of Tarragona, Spain. Participants were recruited at their primary care facilities during their initial prenatal appointments with midwives. The inclusion criteria included healthy women over 18 years old within the first 12 weeks of gestation, without anemia, and with the capacity to comprehend the local and official languages of the region (Spanish and Catalan). Women were excluded from the study if they: had experienced multiple pregnancies, had used iron supplements in the months preceding the 12th week of pregnancy, or had a history of severe diseases such as immunosuppression or chronic conditions that could impact their nutritional development (e.g., anemia, cancer, diabetes, malabsorption, or liver disease). Detailed information can be found elsewhere [22]. 

### 2.2. Maternal Data Collection

In this study, baseline maternal characteristics were collected through face-to-face interviews at recruitment. These encompassed maternal age, smoking habits, self-reported weight and height, and early pregnancy BMI. Gestational weight gain was calculated as the difference between the weights recorded at the initial and third-trimester appointments. Socioeconomic status was determined based on education and occupation. The adherence to the Mediterranean diet (MedDiet) during pregnancy was evaluated using the relative MedDiet (rMedDiet) score, which ranged from 0 to 18 points, with higher scores signifying a stronger adherence to the MedDiet [23]. Serum n-3 polyunsaturated fatty acids (n-3 PUFA) samples were collected at 12th week and 36th week after fasting in 7.5 mL tubes without anticoagulant. After 30 min of coagulation at room temperature, the serum was separated by centrifugation and stored in 500 μL aliquots at −80 °C. Thawing and processing of the samples were conducted simultaneously at the study’s end to minimize batch variation [22]. Serum n-3 PUFA at the 36th week was used due to the recognized predominant transfer of n-3 PUFA from maternal circulation to the fetus during the third trimester [24,25]. Biochemical data (including serum ferritin, serum VitB12, and serum VitD) at the 36th week were utilized for analysis. The red-blood-cell folate (RBC folate) was detected at the 12th week of gestation. VitB12, VitD, and RBC folate concentrations were determined using a competitive immunoassay with direct chemiluminescence technology (ADVIA Centaur, Siemens Healthineers, Madrid, Spain), serum ferritin (μg/mL) was determined using the immunochemiluminescence method. Maternal psychological distress was evaluated using the State–Trait Anxiety Inventory in both the first and third trimesters, and the scores were averaged to represent distress levels across the entire gestational period [26].

A self-administered 45-item food frequency questionnaire (FFQ), specifically validated for our study population, was used to evaluate average dietary intake at weeks 12, 24, and 36 of pregnancy. During this assessment, fish (white fish, blue fish, and seafood) consumption was estimated. The daily diet toxicants (including As, Inorganic As (InAs), cadmium (Cd), MeHg, lead (Pb), polychlorinated dibenzo-p-dioxins and dibenzofurans (PCDD/Fs), DL-PCBs, and non-DL-PCBs (NDL-PCBs)) from fish in our population was estimated using the database of toxicants in food from the Catalan Food Safety Agency, as has been reported in a previously published paper [11]. More detailed information about the methods and results of the study can be found in the Catalan Food Security Agency report, which is publicly available [27]. Pregnant women were categorized according to their fish intake in relation to the Spanish guideline recommendations into <54 g/day (lower than the recommended intake), 54 to 71 g/day (recommended intake) and >71 g/day (exceeding the recommended intake) [28]. 

### 2.3. Infant Data Collection

At around 40 days of age, trained psychologists administered the Bayley Scales of Infant and Toddler Development 3rd edition (BSID-III) [29], which is considered to be the gold standard and is used by clinicians and researchers to assess the developmental functioning of young children [30,31]. BSID-III assessed cognitive, language and motor domains in children aged 1–42 months. The language and motor scales include two subscales: the language scale assesses receptive and expressive language abilities, and the motor scale assesses both fine and gross motor skills. The raw scores for each scale were standardized to a mean of 100 with a standard deviation of 15 and for subscales to a mean of 10 and SD of 3. The assessment with the infant was approximately 30–50 min.

### 2.4. Statistical Analyses

Before conducting analyses, mean imputation was employed in variables with less than 4% of missing values [32]. Subsequently, multiple imputation by fully conditional specification was performed, generating five imputed datasets to address missing data. Linear regression was selected as the type of imputation model, encompassing all confounder variables utilized in the fully adjusted model, along with all exposures and outcomes in the imputation model. The chi-square test and the Kruskal–Wallis test were conducted, as appropriate, to compare descriptive characteristics of the study participants and dietary toxicant intakes from fish, according to the categories of fish consumption amounts. Single-exposure models were performed, employing multivariate linear regressions to assess the association between each exposure (toxicant) and each outcome independently. These models were adjusted for covariates including age, BMI, gestational weight gain, social class, smoking status, MedDiet during pregnancy, energy intake during pregnancy, serum n-3 PUFA, red-blood-cell folate, serum ferritin, serum VitB12, serum VitD, supplementary iron, state–trait anxiety inventory score, newborn sex, newborn weight, and the type of feeding. In the linear regression, we computed β coefficient for a one-unit change in the continuous exposure variables. However, some toxicants showed either high or low median exposure. For instance, arsenic (As) had a median exposure of 274.20 μg/d, while inorganic arsenic (InAs) had a median exposure of 0.05 μg/d. To standardize, we applied a 10-unit change for continuous exposure variables with higher means (As and NDL-PCBs), and 0.01-unit and 0.1-unit changes for those with lower means (InAs and Pb). Multivariate linear regression analysis, adjusted for the same variables as before, was also performed to assess the association between fish consumption amount in three categories and children neurodevelopment at 40 days. In this case, lower than the recommended intake was used as the reference category. Statistical significance was defined as *p* < 0.05. All statistical analyses were conducted with SPSS, version 28.0 (IBM Corp., Armonk, NY, USA).

## 3. Results

### 3.1. Study Design

**Figure 1:**
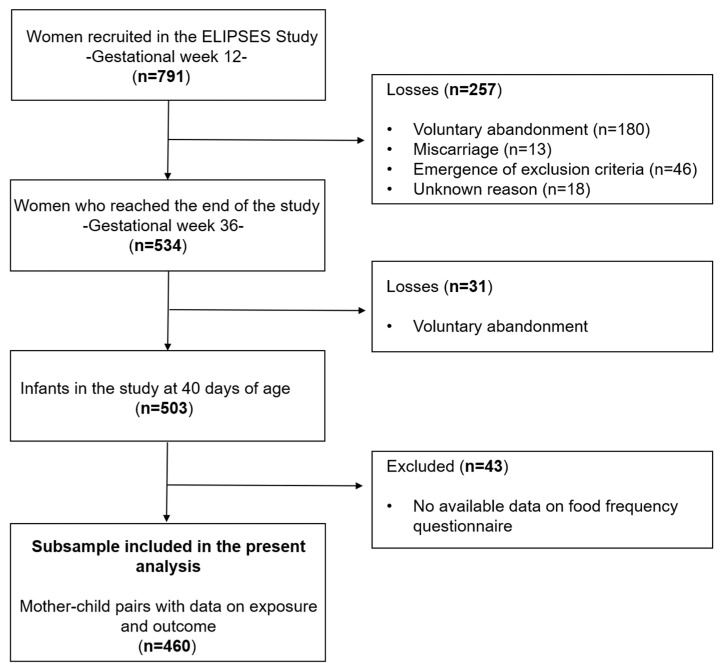
Flowchart of study.

Among the initial 791 recruited women, 534 women completed the study. Then, neurodevelopment was assessed in 503 infants. Out of these, 460 mother–child pairs had information on both the maternal food frequency questionnaire and the assessments of infant neurodevelopment included in the present study (Figure 1).

### 3.2. Characteristics of Participants

From a total of 460 mother–child pairs, the sociodemographic data, lifestyle, and diets of the mother and the psychological and characteristics of the children are presented in Table 1. Briefly, the mean age of the mother was 30.89 ± 5.10 years with older ages in the group of higher fish consumption (*p* < 0.05). No significant differences were found in BMI status, mean gestational weight gain, socioeconomic level, and smoking status by fish consumption. The MedDiet score during pregnancy was 9.85 ± 2.46 and the mean of energy intake was 2041.28 ± 605.77 kcal/day, with higher means for the higher fish consumption category. The mean serum n-3 PUFA level was 258.36 ± 82.78 μmol/L. No differences in micronutrient levels were observed based on the fish consumption categories. Most baseline characteristics (BMI, gestational weight gain, smoking status, MedDiet score, energy intake, biochemical data, iron supplementary, and state–trait anxiety inventory score) did not show significant differences between the pregnant women included in the analysis and those excluded, except for age (30.89 vs. 29.54 years, *p* < 0.01). In terms of social classes, the included group had a lower percentage in the low/middle class (80.9% vs. 86.4%) and a slightly higher percentage in the high class (19.1% vs. 13.6%, *p* = 0.042).

Regarding infants, there were 236 (51.3%) males and 224 (48.7%) females. The mean newborn weight was 3298.03 ± 461.23 g. There were no differences in child gender, newborn weight, type of breastfeeding, and BSID-III score at 40 days among the categories of maternal prenatal fish consumption amounts.

### 3.3. Association between Diet Toxicants from Fish and Fish Consumption Amount

Table 2 illustrates the daily toxicant intake from fish including As, InAs, Cd, MeHg, Pb, PCDD/Fs, DL-PCBs, and NDL-PCBs in pregnant women and among categories of fish consumption amount. In pregnant women, As (274.20 vs. 151.37 µg/d) and DL-PCBs (15.28 pg TEQ/d vs. 20.18 pg TEQ/d) intake from fish exceeded the daily general dietary toxicants threshold reported by EFSA [33,34]. Furthermore, the Kruskal–Wallis comparison test showed significant differences among all groups on all analyzed toxicants (all *p* < 0.01). Then, the post hoc test showed that all the analyzed toxicants were significantly higher in those pregnant women with fish consumption > 71 g/d compared to those with fish consumption < 54 g/d and 54–71 g/d. All the investigated toxicants were also higher in those pregnant women with fish consumption within the recommendations (54–71 g/d) than those with a consumption lower than the recommendations (<54 g/d).

### 3.4. Associations between Maternal Toxicant Intake from Fish and Neurodevelopment Data (BSID-III) of 40-Day Newborns 

Table 3 shows the associations between maternal toxicants intake from fish and language development. In a crude model, As, InAs, MeHg, DL-PCBs, and NDL-PCBs were negatively associated with the language scale (all *p* < 0.05). In the subscales, MeHg and DL-PCBs were negatively associated with the receptive language subscale, while As was adversely associated with the expressive language subscale (all *p* < 0.05).

Similar results were observed after adjusting for potential confounders. As, InAs, MeHg, DL-PCBs, and NDL-PCB were negatively related to language scale (all *p* < 0.05). In the subscales, a negative association was also found in MeHg and DL-PCBs with the receptive language subscale (both *p* < 0.05). Additionally, As was also negatively associated with the expressive language subscale (*p* < 0.05). Specifically, regarding the associations of encountered toxicants with language scales, MeHg (β = −0.41) and InAs (β = −0.40) were most strongly associated, followed by DL-PCBs (β = −0.10) and NDL-PCBs (β = −0.12), with As showing the weakest association (β = −0.06). No associations were found in dietary toxicant intake from fish with motor or cognitive development scales in the crude or adjusted models (Supplementary Table S1).

### 3.5. Association between Maternal Fish Intake and Neurodevelopment Data (BSID-III) of 40 Days Newborns 

Table 4 presents the results of multiple linear regression on the association between fish consumption and language development. Fish consumption exceeding the recommendations (>71 g/d) was related to the lower language scale (*p* = 0.037) but not to the receptive language or expressive language scale (both *p* > 0.05), compared to fish consumption lower than the recommended intake (<54 g/d). However, no association was observed between fish consumption within the recommended intake (54–71 g/d) and any language scale.

After controlling for potential confounding factors, all these associations became stronger. Fish consumption exceeding the recommended intake during pregnancy was associated with the lower language scale (*p* = 0.019) and expressive language subscale (*p* = 0.026). Nevertheless, fish consumption during pregnancy within the recommended intake was not associated with any improvement in, or worsening of, the language scale. After removing serum n-3 PUFA, the results were essentially the same.

Fish consumption was not associated with motor or cognitive development scores in the crude or adjusted models (Supplementary Table S2).

## 4. Discussion

To the best of our knowledge, this is the first study evaluating associations between estimated prenatal dietary fish consumption and derived toxicants with neurodevelopment outcomes at 40 days of age. In this study, dietary toxicants derived from fish consumption, such as As, InAs, MeHg, DL-PCBs, and NDL-PCBs, were associated with worse language development in infants, with no impact on their motor or cognitive development. Furthermore, infants from mothers exceeding the Spanish recommendations of fish consumption had poorer language functions, but motor and cognitive development remained unaffected. These findings highlight the complex interplay between estimated prenatal toxicant exposure and fish consumption in influencing infant neurodevelopment, emphasizing the need for further research to guide maternal health recommendations and food safety policy decisions.

The association between maternal exposure to toxicants and offspring neurodevelopment has been widely discussed over decades, with the exposure primarily assessed through blood and/or hair [35,36,37,38,39,40]. However, studies evaluating the relationship between estimated maternal dietary toxicants in pregnancy and offspring neurodevelopment are still limited. Recent studies conducted in the framework of the Norwegian Mother and Child Cohort Study (MoBa) have described high maternal prenatal dietary MeHg exposure from fish and the maternal general diet, with poor language functions among 3- and 5-year-old children [16,18]. In the same cohort, a negative association was observed between prenatal exposure to dioxins and PCBs from the maternal general diet with language functions among 3-year-old children and 5-year-old girls [17,41]. In Tarragona, Spain, a region known for its heavy industrial activity, we have previously observed the presence of a considerable estimated dietary intake of toxicants in pregnant women, with fish as one of the main contributors [11]. However, the relationship between prenatal exposure to dietary toxicants from fish and neurodevelopment among offspring in this area remains unknown. In the current analysis, the intake of As, InAs, MeHg, DL-PCBs, and NDL-PCBs derived from fish was associated with poor language functions among infants at 40 days, which aligns with previous studies [16,17,18,41]. These previous results, and our findings, are not directly comparable to studies that have analyzed prenatal toxicant exposure via blood or hair [35,36,37,38,39,40]. To do so, a conversion to toxicant concentrations in full blood would be needed before any comparison could be made. The potential explanations for the negative association between the prenatal intake of toxicants from fish and language functions could include: (1) As, InAs, and MeHg might interfere with neurotransmitter systems, promote oxidative stress, and cause neuroinflammation, thereby damaging developing neural tissues and affecting brain functions crucial for language abilities [42,43]; (2) DL-PCBs might alter regional brain volume, which might result in neurodevelopmental and language deficits [44]; and (3) NDL-PCBs could disrupt cellular processes in the brain, such as calcium homeostasis, which is crucial for neuron growth, neurotransmitter release, and overall neural health, potentially leading to language deficits [45]. These findings contribute to the broader understanding of environmental factors and early neurodevelopment, shedding light on potential implications for maternal and child health in regions with distinct toxicant profiles in their diets.

The healthy nutritional profile of fish, coupled with the presence of potential toxicants, has led to confusion regarding its contribution to a healthy diet [14]. Consequently, health authorities across European countries have responded by issuing recommendations regarding the quantity of fish consumption [28,46]. In accordance with the existing literature, our findings support that infants born from women consuming the recommended amounts of fish did not display worse neurodevelopment [20,47]. The potential reasons could be that: (1) the toxicant levels derived from moderate fish consumption are not high enough to affect neurodevelopment; and (2) When fish is consumed at a moderate level, the positive impacts of n-3 PUFA from fish might counteract the negative effects of prenatal exposure to toxicants [48,49]. However, prenatal fish consumption exceeding the Spanish recommendations (>71 g/day) was adversely associated with the language functions of infants, even when accounting for potential confounding factors including serum n-3 PUFA. One potential explanation could be that high toxicants from higher fish consumption may have overridden the potential positive effects of n-3 PUFA, traditionally linked to improved language functions in other studies, suggesting that the health benefits of n-3 PUFA might be insufficient to counteract the adverse impacts of toxicants [49]. Nevertheless, the studies from Norway (>400 g/week, approximately equivalent to 57 g/day, recommended by Norwegian Directorate of Health), USA (>340 g/week, approximately equivalent to 49 g/day, recommended by US Federal Government agencies), and Spain (>340 g/week, equivalent to 49 g/day, recommended by US Federal Government agencies) showed that maternal prenatal fish intake above the recommended limit was not adversely associated with neurodevelopment among their offspring [18,20,50]. These apparently controversial results could be explained due to: (1) the varying types of fish consumed, differing levels of toxicants in fish, and diverse recommended fish consumption amounts across countries may result in distinct outcomes regarding the association between maternal prenatal fish consumption and infant neurodevelopment [51]; and (2) Prenatal higher fish consumption might be related to poor language functions in infants but not in children, the effect of prenatal toxicants exposure from fish on offspring might weaken as the child ages increase due to neurodevelopmental resilience and the emergence of other predominant factors; thus, studies from the same region may yield different outcomes if the age of the subjects under investigation varies.

The major strengths of the study are as follows: (1) Dietary intake during pregnancy was estimated by averaging the information collected in a self-administered 45-item or FFQ collected at weeks 12, 24, and 36 of gestation rather than at a single time-point; (2) The prospective study design, coupled with a validated FFQ administered during pregnancy, and a considerable cohort size, allow for the inclusion of multiple potential confounding variables that could impact child development; and (3) The neurodevelopment outcomes of infants were obtained by trained psychologists through a scale administered to the infant, the BSID III, which has good psychometric properties and is considered the gold standard of neurodevelopmental assessment in young children [30,31]. Nevertheless, the study also has the following limitations: (1) National consumption patterns and toxicant levels within fish were different across different nations; hence, caution should be warranted when generalizing current findings across different regions; (2) The estimation of dietary toxicants relied on data extracted from the Catalan Food Security Agency report database, which might not be representative of all Spanish foods; (3) Despite adjusting for multiple potential confounding variables, due to the observational nature of the study, residual confounding could not be ruled out.

## 5. Conclusions

In summary, specific estimated toxicant intake from fish during pregnancy, and prenatal fish consumption exceeding the recommended intake, are associated with poorer language functions in infants. This highlights the importance of vigilant monitoring of environmental toxicants and the provision of dietary guidance for pregnant women. These findings carry potential implications for both public health strategies and the understanding of child development.

## Figures and Tables

**Table 1:** General characteristics of mother and offspring: sociodemographic data, lifestyle, diet, and psychological characteristics.

		Seafood Consumption			
Characteristics	Total (n = 460)	<54 g/d (n = 318)	54 to 71 g/d (n = 66)	>71 g/d (n = 76)	
Maternal Characteristics	Summary Statistics				*p*
Age (years), mean ± SD	30.89 ± 5.10	30.53 ± 5.09 ^a^	30.85 ± 4.92	32.45 ± 5.03 ^a^	**0.012**
BMI (kg/m^2^), n (%)					0.166
<25 (normal weight)	276 (60.0%)	200 (62.9%)	37 (56.1%)	39 (51.3%)	
25–29 (overweight)	119 (25.9%)	81 (25.5%)	16 (24.2%)	22 (28.9%)	
≥30 (obesity)	65 (14.1%)	37 (11.6%)	13 (19.7%)	15 (19.7%)	
Gestational weight gain (kg), mean ± SD	10.30 ± 4.04	10.35 ± 4.04	10.67 ± 3.61	9.81 ± 4.37	0.429
Social class, n (%)					0.956
Low/middle	372 (80.9%)	256 (80.5%)	54 (81.8%)	62 (81.6%)	
High	88 (19.1%)	62 (19.5%)	12 (18.2%)	14 (18.4%)	
Smoking status, n (%)					0.625
Never/Ex-smoker	390 (84.8%)	273 (85.8%)	54 (81.8%)	63 (82.9%)	
Smoker	70 (15.2%)	45 (14.2%)	12 (18.2%)	13 (17.1%)	
MedDiet during pregnancy (score), mean ± SD	9.85 ± 2.46	9.48 ± 2.43 ^ab^	10.43 ± 2.43 ^a^	10.90 ± 2.20 ^b^	**<0.001**
Energy intake during pregnancy (kcal/d), mean ± SD	2041.28 ± 605.77	1960.29 ± 531.05 ^a^	2093.00 ± 676.29 ^b^	2335.26 ± 734.65 ^ab^	**<0.001**
Serum total n-3 PUFA (μmol/L), mean ± SD	258.36 ± 82.78	251.54 ± 81.11	281.63 ± 91.44	264.94 ± 78.33	0.051
Red blood cell folate (nmol/L), mean ± SD	570.19 ± 209.23	562.36 ± 202.70	549.94 ± 234.78	623.03 ± 213.45	0.148
Serum ferritin (microgr/L), mean ± SD	16.25 ± 9.67	16.20 ± 9.05	17.47 ± 13.64	15.38 ± 7.86	0.523
Serum VitB12 (pg/mL), mean ± SD	305.13 ± 138.95	374.05 ± 135.21	318.59 ± 108.11	310.96 ± 148.07	0.675
Serum VitD (ng/mL), mean ± SD	14.38 ± 6.83	14.61 ± 6.49	14.60 ± 8.70	13.31 ± 6.31	0.429
Iron supplement (mg/day), mean ± SD	49.65± 22.07	49.62 ± 22.39	48.48 ± 21.36	50.79 ± 21.53	0.825
State–trait anxiety inventory score, mean ± SD	16.14 ± 7.21	16.27 ± 7.28	15.09 ± 6.07	16.47 ± 7.83	0.435
Newborn characteristics	Summary statistics				
Sex, n (%)					0.846
Male	236 (51.3%)	161 (50.6%)	36 (54.5%)	39 (51.3%)	
Female	224 (48.7%)	157 (49.4%)	30 (45.5%)	37 (48.7%)	
Newborn weight (g), mean ± SD	3298.03 ± 461.23	3285.39 ± 468.34	3264.36 ± 451.75	3380.13 ± 435.25	0.224
Type of feeding, n (%)					
Breastfeeding	339 (73.7%)	229 (72.0%)	51 (77.3%)	59 (77.6%)	
Mixed feeding/infant formula	121 (26.3%)	89 (28.0%)	15 (22.7%)	17 (22.4%)	0.471
Neurodevelopment of infants	Summary statistics				
BSID-III at 40 days, mean ± SD					
Language scale	96.06 ± 8.27	96.31± 8.03	97.00 ± 8.31	94.12 ± 9.02	0.070
Receptive language	10.59 ± 2.12	10.64 ± 2.05	10.71 ± 2.01	10.28 ± 2.48	0.352
Expressive language	8.02 ± 1.56	8.06 ± 1.59	8.23 ± 1.57	7.68 ± 1.42	0.086
Motor scale	107.47 ± 11.40	106.86 ± 11.69	108.77 ± 10.56	108.91 ± 10.81	0.225
Fine motor	11.45 ± 1.95	11.42 ± 1.90	11.45 ± 2.02	11.53 ± 2.12	0.919
Gross motor	11.06 ± 2.35	10.89 ± 2.38	11.48 ± 2.21	11.42 ± 2.29	0.060
Cognitive scale	101.58 ± 8.78	101.38 ± 9.04	103.11 ± 6.84	101.08 ± 9.10	0.301

*p*-Value for comparisons between categories was calculated by Pearson’s chi-square test or one-factor ANOVA tests for categorical variables and continuous variables, respectively. ^a^, ^b^: <0.05, conducted by post-hoc Bonferroni after ANOVA. Abbreviations: MedDiet, Mediterranean diet; BMI, early pregnancy Body Mass Index; BSID-III, Bayley Scale of Infant Development III. Results in bold are statistically significant.

**Table 2:** Toxicants in fish during pregnancy.

Daily Toxicants	Reference EFSA Values (TWI/TDI)	Comparable Daily EFSA Value ^#^	Toxicants Intake from Total Fish in Total Sample Size (n = 460)	Total Fish Consumption < 54 g/d (n = 318)	Total Fish Consumption 54 to 71 g/d (n = 66)	Total Fish Consumption > 71 g/d (n = 76)	
			Median (IQR)	Median (Q1–Q3)	Median (Q1–Q3)	Median (Q1–Q3)	*p*
As (μg)	15 µg/kg bw/w	151.37	245.74 (190.58)	**194.24 (131.77–264.21) ^ab^**	**366.61 (320.75–406.41) ^ac^**	**505.38 (462.21–581.38) ^bc^**	**<0.001**
InAs (μg)	0.3 µg/kg bw/d	21.19	0.04 (0.03)	**0.03 (0.02–0.04) ^ab^**	**0.06 (0.06–0.06) ^ac^**	**0.08 (0.08–0.10) ^bc^**	**<0.001**
Cd (μg)	2.5 µg/kg bw/w	25.23	2.82 (2.77)	**2.40 (1.13–3.48) ^ab^**	**3.81 (2.67–5.49) ^ac^**	**4.55 (2.42–7.65) ^bc^**	**<0.001**
MeHg (μg)	1.3 µg/kg bw/w	13.12	3.82 (2.81)	**3.05 (2.05–3.93) ^ab^**	**5.47 (4.92–6.12) ^ac^**	**7.88 (6.89–9.09) ^bc^**	**<0.001**
Pb (μg)	0.50 µg/kg bw/d	35.32	0.60 (0.51)	**0.49 (0.28–0.68) ^ab^**	**0.84 (0.69–1.08) ^ac^**	**1.14 (0.77–1.46) ^bc^**	**<0.001**
PCDD/Fs (pg TEQ)	2 pg/kg bw/w *	20.18	2.89 (1.98)	**2.33 (1.69–2.94) ^ab^**	**4.15 (3.81–4.57) ^ac^**	**5.73 (5.02–6.76) ^bc^**	**<0.001**
DL_PCBs (pg TEQ)			13.79 (10.79)	**11.09 (7.38–14.17) ^ab^**	**20.16 (17.03–23.14) ^ac^**	**29.25 (24.04–32.74) ^bc^**	**<0.001**
NDL_PCBs (ng TEQ)	10 ng/kg bw/d	706.40	137.28 (103.02)	**113.01 (75.72–140.86) ^ab^**	**201.18 (190.49–219.12) ^ac^**	**280.12 (253.14–332.33) ^bc^**	**<0.001**

The *p*-value for comparisons between categories was calculated by the Kruskal–Wallis test. ^a^, ^b^, ^c^: <0.05, conducted by the Mann–Whitney U test after the Kruskal–Wallis test. ^#^, TWI/7 × average weight (70.64 kg) or TDI × average weight (70.64 kg); *, 2 pg/kg bw/week is the tolerable weekly intake for both PCDD/Fs and DL-PCBs; TWI, tolerable weekly intake; TDI, tolerable daily intake; As, arsenic; InAs, inorganic arsenic; Cd, cadmium; MeHg, methylmercury; Pb, lead; PCDD/Fs, polychlorinated dibenzo-p-dioxins and dibenzofurans; DL-PCBs, dioxin-like polychlorinated biphenyls; NDL-PCBs, non-dioxin-like polychlorinated biphenyls. IQR, interquartile range. Results in bold are statistically significant.

**Table 3:** Beta-coefficient and 95% confidence interval for the association between maternal toxicants intake from fish and language development of 40-day newborns.

	Language Scale				Receptive Language Subscale				Expressive Language Subscale			
	β	95%CI		*p*	β	95%CI		*p*	β	95%CI		*p*
**As ^a^**												
crude	**−0.05**	**−0.10**	**0.00**	**0.040**	−0.01	−0.02	0.01	0.220	**−0.01**	**−0.02**	**0.00**	**0.045**
Model 1	**−0.06**	**−0.12**	**−0.01**	**0.022**	−0.01	−0.02	0.01	0.223	**−0.02**	**−0.23**	**−0.01**	**0.016**
**InAs ^b^**												
crude	**−0.33**	**−0.64**	**−0.02**	**0.035**	−0.07	−0.15	0.01	0.097	−0.05	−0.10	0.01	0.126
Model 1	**−0.40**	**−0.75**	**−0.06**	**0.020**	−0.08	−0.17	0.01	0.090	−0.06	−0.12	0.01	0.077
**Cd**												
crude	0.04	−0.26	0.34	0.811	0.04	−0.04	0.11	0.369	−0.02	−0.08	0.04	0.452
Model 1	0.03	−0.28	0.34	0.871	0.04	−0.04	0.12	0.383	−0.03	−0.09	0.03	0.379
**MeHg**												
crude	**−0.34**	**−0.66**	**−0.04**	**0.028**	**−0.08**	**−0.17**	**−0.01**	**0.031**	−0.03	−0.09	0.03	0.311
Model 1	**−0.41**	**−0.76**	**−0.07**	**0.019**	**−0.10**	**−0.19**	**−0.01**	**0.030**	−0.04	−0.10	0.03	0.262
**Pb ^c^**												
crude	−0.03	−0.21	0.14	0.693	0.01	−0.03	0.06	0.628	−0.02	−0.06	0.01	0.184
Model 1	−0.05	−0.23	0.13	0.614	0.01	−0.04	0.06	0.640	−0.03	−0.06	0.01	0.127
**PCDD/Fs**												
crude	−0.42	−0.86	0.04	0.071	−0.08	−0.20	0.04	0.168	−0.06	−0.15	0.03	0.174
Model 1	−0.50	−1.00	0.00	0.053	−0.09	−0.22	0.04	0.164	−0.08	−0.17	0.02	0.119
**DL-PCBs**												
crude	**−0.08**	**−0.17**	**0.00**	**0.049**	**−0.02**	**−0.04**	**0.00**	**0.037**	−0.01	−0.02	0.01	0.485
Model 1	**−0.10**	**−0.19**	**−0.01**	**0.040**	**−0.03**	**−0.05**	**−0.01**	**0.036**	−0.01	−0.02	0.01	0.459
**NDL-PCB ^a^**												
crude	**−0.10**	**−0.19**	**−0.01**	**0.031**	−0.02	−0.05	0.00	0.053	−0.01	−0.03	0.01	0.216
Model 1	**−0.12**	**−0.22**	**−0.02**	**0.020**	−0.03	−0.05	0.00	0.051	−0.01	−0.03	0.00	0.161

^a^, 10 units increase; ^b^, 0.01 units increase; ^c^, 0.1 units increase. Abbreviations: As, arsenic; InAs, inorganic arsenic; Cd, cadmium; MeHg, methylmercury; Pb, lead; PCDD/Fs, polychlorinated dibenzo-p-dioxins and dibenzofurans; DL-PCBs, dioxin-like polychlorinated biphenyls; NDL-PCBs, non-dioxin-like polychlorinated biphenyls. Model 1 adjusted by age (years), BMI (normal weight, overweight, obesity), gestational weight gain (kg), social class (low/middle, high) smoking status (never/ex-smoker, smoker), Mediterranean Diet adherence during pregnancy (score), energy intake during pregnancy (kcal/d), total serum n-3 PUFA (μmol/L), red-blood-cell folate (nmol/L), serum ferritin (microgr/L), serum VitB12 (pg/mL), serum VitD (ng/mL), iron supplementary (mg/day), state–trait anxiety inventory (score), newborn gender (male, female), newborn weight (g), type of feeding (breastfeeding, mixed feeding/infant formula). Results in bold are statistically significant.

**Table 4:** Beta-coefficient and 95% confidence interval for the association between maternal fish intake according to Spanish guideline recommendations and language development of 40-day newborns.

		Language Scale				Receptive Language Subscale				Expressive Language Subscale			
Seafood Consumption		β	95%CI		*p*	β	95%CI		*p*	β	95%CI		*p*
<54 g/d (ref.)	Crude												
54–71 g/d		0.69	−1.50	2.88	0.535	0.07	−0.50	0.63	0.814	0.17	−0.25	0.58	0.436
>71 g/d		**−2.19**	**−4.25**	**−0.13**	**0.037**	−0.36	−0.90	0.16	0.174	−0.38	−0.77	0.11	0.057
<54 g/d (ref.)	Model 1												
54–71 g/d		0.40	−1.87	2.66	0.733	0.08	−0.50	0.67	0.781	0.05	−0.38	0.48	0.817
>71 g/d		**−2.70**	**−4.97**	**−0.44**	**0.019**	−0.43	−1.02	0.16	0.151	**−0.49**	**−0.92**	**−0.06**	**0.026**

Abbreviations: d, day; ref., reference. Model 1 adjusted by age (years), BMI (normal weight, overweight, obesity), gestational weight gain (kg), social class (low/middle, high) smoking status (never/ex-smoker, smoker), Mediterranean Diet adherence during pregnancy (score), energy intake during pregnancy (kcal/d), serum n-3 PUFA (μmol/L), red-blood-cell folate (nmol/L), serum ferritin (microgr/L), serum VitB12 (pg/mL), serum VitD (ng/mL), iron supplementary (mg/day), State-trait anxiety inventory (score), newborn gender (male, female), newborn weight (g), type of feeding (breastfeeding, mixed feeding/infant formula). Results in bold are statistically significant.

## Data Availability

The datasets generated and/or analyzed during the study are not publicly accessible due to considerations of subject confidentiality. However, they can be obtained from the corresponding author upon reasonable request.

## Supplementary Materials

The following supporting information can be downloaded at: https://www.mdpi.com/article/10.3390/toxics12050338/s1, Table S1: Beta-coefficient and 95% confidence interval for the association between maternal toxicants intake from fish with motor and cognitive development of 40-day newborns. Table S2: Beta-coefficient and 95% confidence interval for the association between maternal fish intake according to Spanish guideline recommendations and language development of 40 days newborns.
