# Supplementary material for: Association between Prenatal Dietary Toxicants and Infant Neurodevelopment: The Role of Fish
Source: Toxics. 2024 May 6;12(5):338. doi: 10.3390/toxics12050338 (PMC11126097; doi:10.3390/toxics12050338)
Supplement: Supplementary file 1 [file toxics-12-00338-s001.zip › toxics-2980515-supplementary.pdf]

Supplementary table S1. Beta-coefficient and 95% confidence interval for the association between maternal toxicants intake from fish with motor and cognitive development of 40 days newborns

	Motor scale		Fine motor subscale		Gross motor subscale		Cognitive scale	
	β (95%CI)	P	β (95%CI)	P	β (95%CI)	P	β (95%CI)	P
**As**								
crude	0.06 (0.00~0.13)	0.059	0.00 (-0.01~0.01)	0.748	0.01 (-0.01~0.03)	0.193	-0.01 (-0.06~0.04)	0.650
Model 1	0.06 (-0.01~0.14)	0.091	0.00 (-0.02~0.01)	0.696	0.02 (-0.01~0.04)	0.094	-0.03 (-0.08~0.03)	0.363
**InAs**								
crude	0.32 (-0.11~0.75)	0.142	0.00 (-0.07~0.08)	0.920	0.09 (0.00~0.17)	0.057	-0.10 (-0.43~0.23)	0.560
Model 1	0.30 (-0.18~0.77)	0.218	-0.03 (-0.11~0.05)	0.516	0.10 (0.00~0.19)	0.053	-0.20 (-0.56~0.17)	0.297
**Cd**								
crude	0.30 (-0.12~0.71)	0.160	0.02 (-0.05~0.09)	0.541	0.06 (-0.03~0.14)	0.175	0.04 (-0.28~0.36)	0.815
Model 1	0.16 (-0.26~0.58)	0.461	-0.01 (-0.08~0.07)	0.878	0.04 (-0.05~0.13)	0.387	0.00 (-0.33~0.33)	0.985
**MeHg**								
crude	0.20 (-0.24~0.63)	0.371	-0.01 (-0.08~0.07)	0.857	0.06 (-0.03~0.15)	0.194	-0.12 (-0.46~0.21)	0.470
Model 1	0.17 (-0.30~0.65)	0.478	-0.03 (-0.12~0.05)	0.409	0.07 (-0.03~0.16)	0.180	-0.21 (-0.58~0.15)	0.253
**Pb**								
crude	0.22 (-0.02~0.46)	0.070	0.01 (-0.03~0.05)	0.514	0.05 (0.00~0.10)	0.054	0.01 (-0.18~0.19)	0.928
Model 1	1.52 (-0.96~3.99)	0.229	-0.04 (-0.46~0.39)	0.863	0.40 (-0.11~0.91)	0.126	-0.21 (-2.13~1.72)	0.835
**PCDD/Fs**								
crude	0.46 (-0.17~1.08)	0.148	0.01 (-0.10~0.12)	0.881	0.12 (-0.01~0.25)	0.071	-0.12 (-0.60~0.36)	0.621
Model 1	0.38 (-0.30~1.06)	0.272	-0.04 (-0.16~0.08)	0.512	0.12 (-0.02~0.26)	0.086	-0.25 (-0.78~0.28)	0.353
**DL-PCBs**								
crude	0.04 (-0.08~0.16)	0.503	0.00 (-0.02~0.02)	0.813	0.01 (-0.01~0.04)	0.318	-0.03 (-0.12~0.06)	0.486
Model 1	0.03 (-0.10~0.15)	0.670	-0.01 (-0.03~0.01)	0.382	0.01 (-0.01~0.04)	0.335	-0.05 (-0.15~0.04)	0.284
**NDL-PCBs**								
crude	0.07 (-0.05~0.20)	0.248	0.00 (-0.02~0.02)	0.957	0.02 (-0.01~0.05)	0.116	-0.03 (-0.13~0.06)	0.508
Model 1	0.07 (-0.07~0.20)	0.349	-0.01 (-0.03~0.01)	0.446	0.02 (-0.01~0.05)	0.109	-0.06 (-0.17~0.05)	0.269

a, 10 units increase; b, 0.01 units increase; c, 0.1 units increase. Abbreviations: As, arsenic; InAs, inorganic arsenic; Cd, cadmium; MeHg, methylmercury; Pb, lead; PCDD/Fs, polychlorinated dibenzo-p-dioxins and dibenzofurans; DL-PCBs, dioxin-like polychlorinated biphenyls; NDL-PCBs, non-dioxin-like polychlorinated biphenyls.

Model 1 adjusted by age (years), BMI (normal weight, overweight, obesity), gestational weight gain (kg), social class (low/middle, high) smoking status (never/ ex-smoker, smoker), Mediterranean Diet adherence during pregnancy (score), energy intake during pregnancy (kcal/d), total serum n-3 PUFA (μmol/L), red blood cell folate (nmol/L), serum ferritin (microgr/L), serum VitB12 (pg/mL), serum VitD (ng/mL), iron supplementary (mg/day), State-trait anxiety inventory (score), newborn gender (male, female), type of feeding (breastfeeding, mixed feeding/infant formula).

Supplementary table S2. Beta-coefficient and 95% confidence interval for the association between maternal fish intake according to Spanish guideline recommendations and language development of 40 days newborns

		Motor scale		Fine motor subscale		Gross motor subscale		Cognitive scale	
Seafood consumption		β (95%CI)	P	β (95%CI)	P	β (95%CI)	P	β (95%CI)	P
<54 grams/d (ref.)									
54-71 grams/d	Crude	1.91 (-1.11~4.93)	0.214	0.03 (-0.49~0.55)	0.910	0.60 (-0.03~1.22)	0.060	1.73 (-0.60~4.05)	0.146
>71 grams/d		2.05 (-0.80~4.90)	0.159	0.10 (-0.39~0.59)	0.683	0.53 (-0.06~1.12)	0.076	-0.30 (-2.50~1.89)	0.786
<54 grams/d (ref.)									
54-71 grams/d	Model 1	2.07 (-1.04~5.18)	0.193	-0.03 (-0.56~0.50)	0.919	0.70 (-0.06~1.33)	0.053	1.87 (-0.54~4.28)	0.128
>71 grams/d		1.69 (-1.43~4.81)	0.288	-0.09 (-0.63~0.44)	0.733	0.54 (-0.10~1.19)	0.097	-0.76 (-3.18~1.66)	0.539

Abbreviations: d, day; ref., reference.

Model 1 adjusted by age (years), BMI (normal weight, overweight, obesity), gestational weight gain (kg), social class (low/middle, high) smoking status (never/ ex-smoker, smoker), Mediterranean Diet adherence during pregnancy (score), energy intake during pregnancy (kcal/d), total serum n-3 PUFA (μmol/L), red blood cell folate (nmol/L), serum ferritin (microgr/L), serum VitB12 (pg/mL), serum VitD (ng/mL), iron supplementary (mg/day), State-trait anxiety inventory (score), newborn gender (male, female), type of feeding (breastfeeding, mixed feeding/infant formula).
